# hg19K: addressing a significant lacuna in hg19‐based variant calling

**DOI:** 10.1002/mgg3.251

**Published:** 2016-11-13

**Authors:** Savita Karthikeyan, Pushpinder S. Bawa, Subhashini Srinivasan

**Affiliations:** ^1^ Institute of Bioinformatics and Applied BiotechnologyBiotech Park, Electronic City Phase IBangalore560100India

**Keywords:** 1000 genomes project, alternate reference, hg19, major allele, minor allele

## Abstract

**Background:**

The hg19 assembly of the human genome is the most heavily annotated and most commonly used reference to make variant calls for individual genomes. Based on the phase 3 report of the 1000 genomes project (1000G), it is now well known that many positions in the hg19 genome represent minor alleles. Since commonly used variant call methods are developed under the assumption that hg19 reference harbors major alleles at all the ~3 billion positions, these methods mask the calls whenever an individual is homozygous to the minor allele at the respective positions. Hence, it is important to address the extent and impact of these minor alleles in hg19 from the point of view of individual genomes.

**Method:**

We have created a reference genome, hg19K, in which all the positions in hg19 reference harboring minor allele were replaced by those from the phase 3 report of the 1000 genomes project. The genomes of five individuals, downloaded from the public repository, were analyzed using both hg19 and hg19K and compared.

**Results:**

Out of the 81 million SNPs in phase 3 report from the 1000 genomes project, 1.9 million positions were found to be major alleles compared to hg19 with many having an allele frequency of >0.9. We observed that ~30% of the SNVs found in individual genomes are confined to the 1.9 million positions. Also, there are ~8% unique SNVs predicted using hg19K‐based approach, which are also confined to the 1.9 million positions.

**Conclusion:**

We report that the presence of minor alleles in hg19 alone results in ~8% false negatives and ~30% false positives during variant calls. Also, among the variant calls unique to hg19K‐based methods, which are missed in individuals homozygous to the minor alleles in hg19‐based prediction, some are deleterious missense mutations at sites conserved across diverse species.

## Introduction

The version of the human genome assembly, hg19, is the most widely used and the most heavily annotated human genome to date and continues to serve as a strong foundation toward deciphering the role of gene expression, genetic variation, disease predisposition and population diversity. The hg19 assembly is now routinely used in profiling variants from genomes of individuals for use in forensics, diagnostics, genetic disorders, and disease management. The underlying assumption being that hg19 harbors major alleles at all 3 billion positions. However, as more and more genomes of individuals from diverse ethnicity is sequenced, such as the 1000 genomes project, it is becoming clear that significant positions on the hg19 reference assembly, which is the mosaic of genomes from six diverse individuals (Venter et al. 2001), do not represent major alleles at all three billion positions. This scenario is changing as genomes of more individuals are sequenced.

The 1000 genomes project was launched in 2008 to identify and catalog all SNPs in the human population (1000 Genomes Project Consortium et al. 2015). The individuals nominated for sequencing include individuals selected from 26 different geographical locations around the globe weighted by the size of the populations. By the close of the project in 2015, genomes and exomes of 2692 individuals from diverse ethnicity were sequenced, analyzed, and results made available to the public repositories. Not surprisingly, many of the alleles in hg19 turned out to be minor allele based on the variant calls from 1000 genomes project.

The concept of major allele reference genomes representing different ethnic populations has been proposed (Dewey et al. 2011). Here, the authors have used the major alleles from 1000 genomes project to create ethnically concordant reference sequence to improve genotype accuracy. They also show how ethnicity‐based approaches can help interpret genetic variation in the context of disease‐risk prediction. However, the impact of minor alleles in hg19 on the ongoing efforts to variant calling from individual's genomes is not yet addressed.

The major assumption in any variant calling method a is that the reference genome harbors major alleles at all the three billion positions on the human chromosomes. In other words, positions where individual genomes are homozygous to the alleles present in the reference genome carries no information about the respective individuals, ethnicity, ancestry, predisposition, or their disease condition. Under this assumption, minor alleles in the reference genome will mask reporting of the minor alleles present in individual genomes, resulting in false negatives. The extent of false negatives will directly depend on the number of minor alleles among the three billion positions in the hg19 assembly and the extent of diversity at these positions within a given genome or population. Here, we address the extent of false discovery in variant calling approaches commonly used.

## Materials and Methods

The hg19 reference sequence, as used by the 1000 genomes project, was downloaded directly from the 1000 genomes ftp repository (ftp://ftp-trace.ncbi.nih.gov/1000genomes/ftp/technical/reference/). The variant file from the phase 3 of the 1000 Genomes Project was downloaded from the 1000 genomes project website (ftp://ftp.1000genomes.ebi.ac.uk/vol1/ftp/release/20130502/). The coordinates in the 1000 Genomes variant call‐set that had an overall alternate allele frequency of more than 0.5 were extracted. These positions were changed in hg19 with the major allele in these positions from the 1000 genomes project using GATK's AlternateReferenceMaker tool to create a new reference called hg19K. Positions that had multiple alternate alleles were discarded.

Whole genome sequences from five individuals from ERP006077 dataset in the public repository were selected based on similar depth of sequencing. These samples are from prostate tissues of individuals with cancer. Samples were mapped onto both hg19 and hg19k using Bowtie2, duplicates were removed using Picard Tools and single‐nucleotide variants were called using SAMtools mpileup and BCFtools. The variant calls were filtered by read depth of at least five and a variant quality of at least 30. The filtered set of SNPs for each sample was annotated against the reference sequences using the SnpEff software (Cingolani et al. 2012). For annotating against hg19, we used the database available with SnpEff. However, for annotating the SNVs from hg19k, a new database for SnpEff was built using the hg19K genome. The hg19K reference and the associated SnpEff database are available upon request to the corresponding author.

For validation of the extent of false negatives using hg19 as reference in other ethnicity with normal biology, we have repeated the pipeline on genome sequence of similar depth from normal samples of two Indians from a remote corner of Karnataka state. Also for these samples, we used modified pipeline that replaces mapping by BWA instead of Bowtie2.

## Results

**Figure 1:**
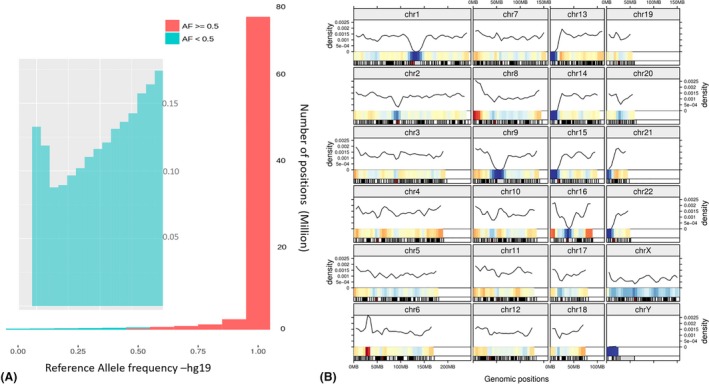
Provides statistics of the SNPs and major alleles from the 1000 genomes project. (A) shows the distribution of allele frequencies in hg19 corresponding to the 81 million SNPs reported by phase 3 of the 1000 genomes project with cyan showing allele frequencies <0.5. Inset shows the enlarged view of alleles <0.5. (B) Show the distribution of the 1.9 million minor alleles across the hg19 sequence.

According to the genomes and exomes of 2692 individuals, sequenced, analyzed, and reported as part of the 1000 genomes project, there are 81 million SNPs compared to the hg19 reference, out of which hg19 harbors minor alleles at 1.9 million positions. Figure 1 shows the allele frequencies and distribution of the 1.9 million variants in the hg19 reference. Figure 1A shows both the overall distribution of allele frequencies at the 81 million positions (red) and the frequency distribution of the 1.9 million minor alleles in hg19 (inset). The number of minor alleles with frequencies below 0.5 in hg19 is uniformly distributed across various frequency levels. For example, the number of minor alleles in hg19 with allele frequencies between 0 and 0.1 is 374607, between 0.1 and 0.2 is 2856603, between 0.2 and 0.3 is 340144, between 0.3 and 0.4 is 400727, and between 0.4 and 0.5 is 479231. In Figure 1B the distribution of these 1.9 million SNPs across the human chromosomes is shown. Except in regions marked blue (low density) and red (high density) in the bottom of each chromosome in Figure 1B, the 1.9 million minor alleles are distributed uniformly over the lengths of all the chromosomes.

Using current approaches for variant calling using hg19, it is not possible to profile the 1.9 million minor alleles across population because variations are called with respect to the hg19 alleles. To demonstrate the significance of the 1.9 million minor allele positions from the point of view of individual genomes, we have created a reference genome, hg19K, that replaces the alleles at the 1.9 million positions in the hg19 reference with the major alleles at the respective positions as reported in phase 3 of the 1000 genomes project.

**Figure 2:**
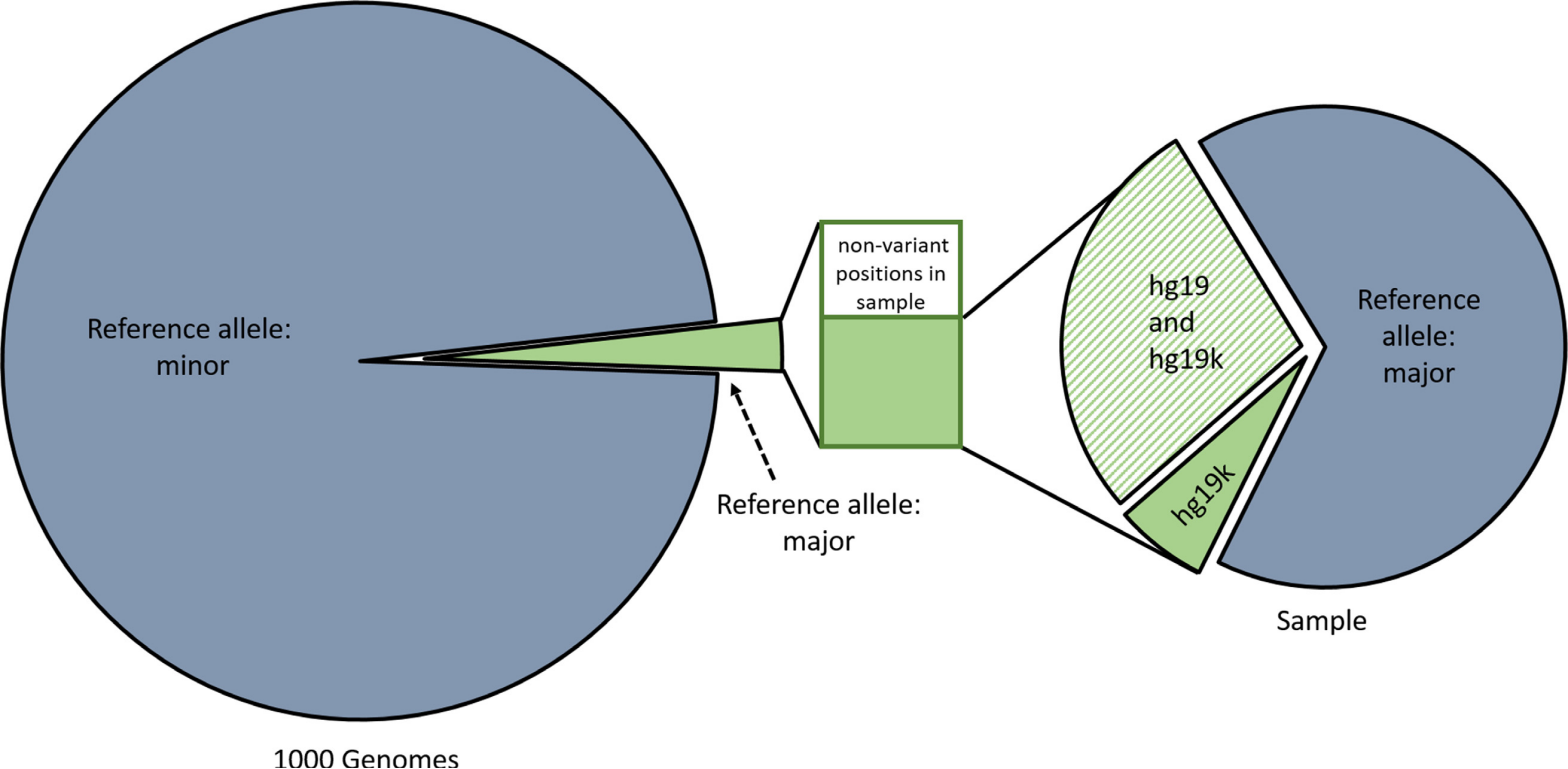
Provides a view of the distribution of SNPs and variants. The Venn to the left shows the proportion of major alleles within the 81 million SNPs reported in phase 3 of the 1000 genomes project. The Venn to the right shows the fraction of these in an average individual sample.

Using both hg19 and hg19K as reference genomes, variant calls for genomes from five individuals, downloaded from the public repository, were performed. These samples were from prostate tissues of individual of undisclosed ethnicity diagnosed with cancer. Around 3.54 (±0.03) million and 2.71 (±0.02) million variant calls are predicted for the five genomes using hg19 and hg19K, respectively. As shown in Figure 2 and Table 1, a large number of these calls (~2.47 ± 0.03 million) were commonly predicted using both references. Among these positions, using both the reference genomes, ~20% (percentage computed for columns 7 with respect to 4 in Table 1) were at positions common to both predictions. These positions are where hg19 harbors minor alleles (1.9 million) and were heterozygous in respective samples. The ~1 million variants calls uniquely predicted using hg19 (column 8 in Table 1) overlap with the 1.9 million minor allele positions. These are actually the major alleles at these positions according to the 1000 genomes project, and are clearly false positives in variant calls using hg19 as reference. In other words, there is over‐representation of variant in hg19‐based calling at these positions burdening downstream analysis and annotation. On the other hand, majority of the ~0.25 million positions (column 6 of Table 1) uniquely predicted by hg19K‐based calling and missed by hg19, are at positions where individuals are homozygous to the minor alleles in hg19. These SNVs are false negatives in hg19‐based prediction.

**Table 1:** Comparative number of variants called per sample

Samples	Number of variants called					Overlap with 1.9 m positions			Percentage	
	Total calls (hg19)	Total calls (hg19k)	Common to both hg19 and hg19k	Unique to hg19	Unique to hg19k	Common to both hg19 and hg19k	Unique to hg19	Unique to hg19k	False positives	False negatives
T2	3523566	2706277	2464798	1058768	241479	528792	1054623	231312	29.931	8.547
T5	3576580	2739673	2510844	1065736	228829	533822	1061286	218090	29.673	7.960
T7	3526146	2703089	2465770	1060376	237319	531096	1056067	226438	29.950	8.377
T9	3514195	2688450	2455194	1059001	233256	525819	1054619	222620	30.010	8.281
T18	3566071	2727320	2496670	1069401	230650	533797	1065220	220366	29.871	8.080
S1	3297701	2448217	2198875	1098883	249342	417178	1094338	238850	33.185	9.756
S7	2960779	2149583	1865248	1095531	284335	304256	1090309	273521	36.825	12.724
S1_bwa	3708065	2837119	2587491	1120574	249628	467586	1105687	229644	29.818	8.094
S7_bwa	3646224	2782382	2513539	1132685	268843	432290	1114869	245991	30.576	8.841

Lists number of variant calls using both hg19 and hg19K. First 5 rows in column 1 are samples used as test and last four rows are used for validation. Columns 2–6 are variant calls using the two references along with number of variants common to both (column 4) and unique to the two (column 5–6). Columns 7–9 show overlap of these variants with the 1.9 million minor allele positions in hg19. Last two columns list the percentage of false positive and negative calls per sample using hg19 as reference.

Figure 2 shows the overall percentage of SNVs predicted for the five genomes. The Venn to the left shows minor to major allele distribution within the 81 million SNPs reported by phase 3 of 1000 genomes project, and the Venn on the right shows the distribution of the predicted SNVs from individual genomes using hg19k. According to this, 25% of the SNVs from individual genomes fall within the 2% (1.9 million) of the major alleles contained in the 81 million SNPs reported by phase 3 of the 1000 genomes project. In other words, although hg19K and hg19 only differ at 1.9 million positions, which is only 2% of all SNPs reported by 1000 genomes project, 25% of the individual variations are confined to these positions.

These findings are now validated in normal samples from potentially different ethnicity using both bowtie and BWA. Table 1 compares the results from the five test samples and the two validation samples using both bowtie and BWA. We have shown that irrespective of the mapping tools, ethnicity or disease state, the percentage of false positives and false negatives remain significantly high at ~30 and ~7, respectively using hg19 as reference.

**Figure 3:**
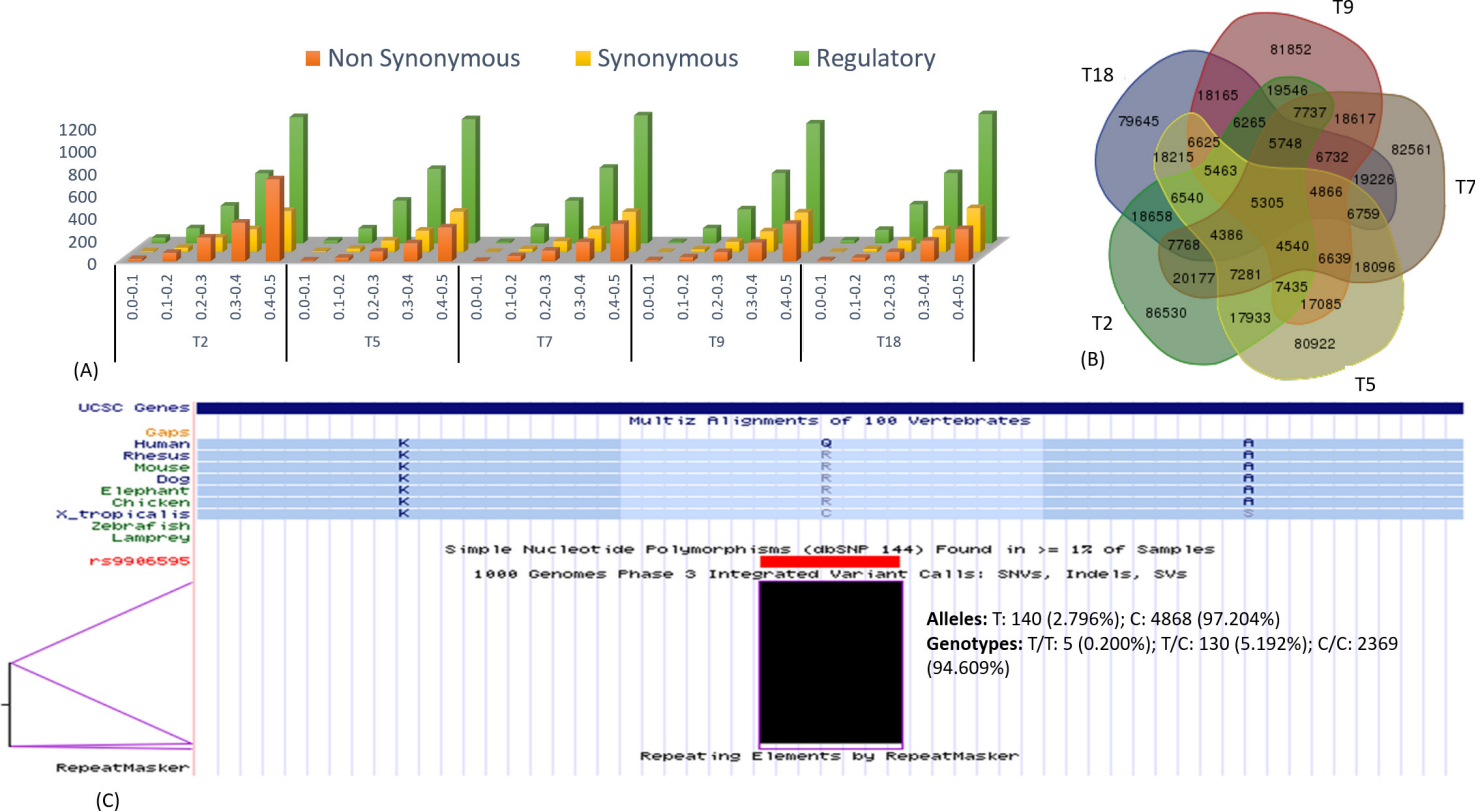
provide some statistic of the distribution of minor/major alleles in samples. (A) Number of alternative alleles in all five samples with frequencies <50% for three different functional class of variants. (B) Show diversity in individual genome within the positions only predicted by hg19K. (C) Snapshot of UCSC browser showing an SNP on hg19, chromosome 17, position 37101380 that is reported as coding for glutamine in hg19.

Figure 3 shows the allele frequencies of the variants in all five samples predicted by hg19K but missed by hg19. As shown in Figure 3A, a number of variants from all five samples have allele frequencies of <50%, which includes some predicted as deleterious with allele frequencies of <0.1. The average number of missense mutations with allele frequency between 0.0 and 0.1 is 10, between 0.1 and 0.2 is 43, between 0.2 and 0.3 is 111, between 0.3 and 0.4 is 204, and between 0.4 and 0.5 is 395. Figure 3B shows diversity in SNVs among the genomes of the five individuals that were unique to hg19K‐based predictions, suggesting the importance of these positions to genetic diversity studies. There are 34 missense mutations reported uniquely by hg19K across the five samples with AAF <0.1 as shown in Table 2. Although these are classified as nonsynonymous mutations using the SnpEff database compatible to hg19K (created in‐house), they are actually the broadly accepted reference allele at those positions in hg19. In four out of the five nonsynonymous mutations reported in Table 2 with allele frequency <3%, the amino acid coded by the reference allele in hg19 suggests deleterious mutation. For example, as shown in Figure 3C, the allele at position chr17:37101380 in hg19 codes for Glutamine, which is not only Arginine in hg19K but Arginine is conserved across species. This is true for majority of the nonsynonymous mutations reported in Table 2. Unfortunately, all the currently used function prediction tools are built with hg19 alleles and hence, could not be used for more systematic function prediction at the 1.9 million minor allele positions. Since the human reference proteome is built using hg19 as reference, the mutations reported here has relevance to the study of human biology in general.

**Table 2:** Nonsynonymous mutations unique to hg19K and missed by hg19

chr	Nucleotide substitution	Gene	Amino acid substitution	Alternate allele frequency
chr2	g.130949411T>G	*TUBA3E*	p.(Glu449Ala)	0.020567
chr6	g.29364787T>C	*OR12D2*	p.(Phe104Ser)	0.021166
chr6	g.33382288A>G	*PHF1*	p.(Lys304Arg)	0.022364
chr2	g.231149108A>G	*SP140*	p.(Lys516Glu)	0.026158
chr17	g.37101380C>T	*FBXO47*	p.(Arg209Gln)	0.027955
chr14	g.24901276T>G	*KHNYN*	p.(Leu270Trp)	0.040535
chr16	g.1389153A>C	*BAIAP3*	p.(Thr87Pro)	0.04373
chr19	g.29704010C>A	*UQCRFS1*	p.(Ala6Ser)	0.046126
chr22	g.22989256G>A	*GGTLC2*	p.(Gly70Glu)	0.046925
chr16	g.1370597C>G	*UBE2I*	p.(Ser164Arg)	0.049521
chr16	g.1370614G>C	*UBE2I*	p.(Arg170Thr)	0.049521
chr21	g.37617630G>T	*DOPEY2*	p.(Gly1118Cys)	0.050719
chr9	g.140130606T>A	*SLC34A3*	p.(Val513Glu)	0.053914
chr6	g.29141743G>A	*OR2J2*	p.(Ala111Thr)	0.058506
chr6	g.42666145T>C	*PRPH2*	p.(Lys310Arg)	0.058706
chr2	g.180810264T>A	*CWC22*	p.(Arg773Ser)	0.06869
chr17	g.80895933G>A	*TBCD*	p.(Gly1135Glu)	0.069089
chr2	g.96795857C>T	*ASTL*	p.(Arg222Gln)	0.072883
chr14	g.36789729G>T	*MBIP*	p.(Ser22Arg)	0.073083
chr3	g.12046364C>G	*SYN2*	p.(Pro37Ala)	0.073682
chr6	g.29911256G>T	*HLA‐A*	p.(Glu185Asp)	0.074281
chr11	g.111749349T>A	*FDXACB1*	p.(Asn87Ile)	0.07528
chr2	g.44104925C>T	*ABCG8*	p.(Ala632Val)	0.077077
chrX	g.65382685C>T	*HEPH*	p.(Ala39Val)	0.079205
chr19	g.56047448G>A	*SBK2*	p.(Arg72Cys)	0.082069
chrX	g.88008423C>A	*CPXCR1*	p.(Ser3Try)	0.086358
chr19	g.36497358G>C	*SYNE4*	p.(His278Gln)	0.08726
chrX	g.84563135A>T	*POF1B*	p.(Leu349Met)	0.087947
chr17	g.72938100C>T	*OTOP3*	p.(Pro119Ser)	0.09385
chr5	g.741736T>G	*ZDHHC11B*	p.(Asp314Ala)	0.095248
chr5	g.140559596G>T	*PCDHB8*	p.(Val661Leu)	0.097644
chr1	g.155026942C>A	*ADAM15*	p.(Thr191Lys)	0.098043
chr1	g.34330067C>A	*HMGB4*	p.(Ala92Glu)	0.098842
chr7	g.150500729G>A	*TMEM176A*	p.(Ala122Thr)	0.09984

Lists 34 nonsynonymous mutations with allele frequencies <10%. Column 2 represents the nucleotide substitution as per hg19K reference. Column 3 represents the gene in which the mutation lies, and Column 4 gives the corresponding amino acid substitution. Column 5 is the allele frequency for the alleles in hg19.

## Discussion

The work reported here reveals a lacuna in hg19‐based variant calling of individual genomes, especially in population genetics and in some cases to personalized medicine. As demonstrated here, as high as ~8% of the variants can be missed and ~30% false positives can result from hg19‐based variant calling. Currently, the high levels of false positives overwhelm the downstream analysis. We have created hg19K, a reference genome representing the mosaic of major alleles from both the 1000 genomes project phase 3 variants and hg19. The reference hg19K is far from complete as it only considers replacing SNPs in this version. A more complete reference genome of major alleles would require replacement of major alleles of other types, such as INDELs, CNVs, and translocation reported in the 1000 genomes project. However, replacing these would disturb the coordinate frame of hg19 rendering it less useful.

To the best of our knowledge, this is a first attempt to improve human reference genome horizontally. A reference genome representing the mosaic of all major alleles is a dynamic process. As more and more genomes are sequenced more sites in hg19 will emerge as harboring minor alleles. However, considering that the individuals selected by the 1000 genomes project are from diverse ethnicity from across the world, the major alleles reported by the 1000 genomes project and hg19 together is likely to remain stable over time; making hg19K stable and useful over the foreseeable future.

## Conflict of Interest

The authors have no conflict of interest. Also, the hg19K genome will be available upon request from the corresponding author.
